# Valuing EQ-5D-Y: the current state of play

**DOI:** 10.1186/s12955-022-01998-8

**Published:** 2022-07-06

**Authors:** N. Devlin, T. Pan, S. Kreimeier, J. Verstraete, E. Stolk, K. Rand, M. Herdman

**Affiliations:** 1 Centre for Health Policy, Melbourne School of Population and Global Health, University of Melbourne, Level 4, 207 Bouverie St, Parkville, VIC 3010 Australia; 2 Office of Health Economics, London, UK; 3 Department of Health Economics and Health Care Management, Faculty of Health Science, Bielefeld University, Bielefeld, Germany; 4 Division of Medicine, Department of Paediatrics and Child Health, University of Cape Town, Cape Town, South Africa; 5 EuroQol Research Foundation, Rotterdam, Netherlands; 6 Health Services Research Centre, Akershus University Hospital, Nordbyhagen, Norway

**Keywords:** Paediatric, HRQoL, EQ-5D-Y, Utilities, Values, DCE, TTO, QALYs, Children, Adolescents, Stated preferences

## Abstract

**Background:**

For nearly a decade, value sets for the EQ-5D-Y were not available, reflecting challenges in valuing child HRQoL. A methodological research programme led to publication of a valuation protocol in 2020, which was rapidly taken up by local study teams. By the end of 2022, between 11 and 17 EQ-5D-Y value sets will be available, more than for any other child HRQoL measure. It is timely to review the experience of those using the protocol to identify early learnings and remaining issues where more research is needed.

**Methods:**

In June 2021, the EuroQol Group organised a three-day workshop, bringing together all those involved in EQ-5D-Y value set studies and related methodological research concerning EQ-5D-Y and valuation. Workshop discussions were captured by note taking and recording all sessions and online chat. A narrative summary of all sessions was produced and synthesised to identify points of agreement and aspects of methods where uncertainty remains.

**Results:**

There was broad agreement that DCE is working well as the principal valuation method. However, the most appropriate means of anchoring the latent scale values produced by DCE remains unclear. Some studies have deviated from the protocol by extending the number of states included in TTO tasks, to better support modelling of DCE and TTO. There is ongoing discussion about the relative merits of alternative variants of TTO and other methods for anchoring. Very few studies have consulted with local end-users to gauge the acceptability of methods used to value EQ-5D-Y.

**Conclusions:**

Priority areas for research include testing alternative methods for anchoring DCE data; exploring the preferences of adolescents; and scale differences in values for EQ-5D-Y and adult EQ-5D states, and implications of such differences for the use of EQ-5D-Y values in HTA. Given the normative elements of the protocol, engaging with HTA bodies and other local users should be the first step for all future value set studies. Value sets undertaken to date are for the three-level EQ-5D-Y. However, the issues discussed in this paper are equally relevant to valuation of the five-level version of EQ-5D-Y; indeed, similar challenges are encountered valuing *any* measure of child HRQoL.

## Background

Children and adolescents are important users of health care services, often with distinctive health needs. Many new technologies are aimed either at children, or have patient populations which include children and young people. However, decisions about whether new interventions for children are effective and cost effective often encounter substantial gaps in evidence. National Institute for Health and Care Excellence (NICE) technology assessments that have made recommendations about children often involved the use of measures of health-related quality of life (HRQoL) intended for *adults*; few used child-specific HRQoL measures or values [1]. Similar gaps in evidence on child HRQOL have been noted in PBAC submissions [2]. The lack of data on children’s HRQoL is a common feature of Health Technology Assessment (HTA) of paediatric technologies worldwide [3].

A number of child-specific measures of HRQoL are available [4] which in principle could be used to generate the evidence needed for HTA. However, to date, relatively few value sets (i.e., preference-weighted scoring systems) have been available to accompany them. These value sets represent how good or bad health problems are considered to be, based on people’s preferences about health. They allow each state described by a HRQoL measure to be summarised on a scale anchored at 0 (dead) and 1 (full health), as required for their use in estimating quality adjusted life years (QALYs) [5].

The lack of paediatric HRQoL values reflects a number of unresolved questions about how best to value child HRQoL. While methods for valuing adult HRQoL are well established and accepted, the valuation of child HRQoL raises additional issues. Central to these issues is a value judgement about whether it is the preferences of adults or children that are relevant in valuing child HRQoL [6]. Related methods questions concern the perspective adults should be asked to adopt if valuing child health (e.g., imagining experiencing the state themselves; or themselves as a child, or imagining a hypothetical child); what age of child to consider; and how different ways of framing the tasks might influence the values that are produced. An alternative is to seek children’s preferences, asking them to imagine states from their own perspective. However, this raises questions about feasibility, given the cognitive demands of valuation tasks, and ethics, where tasks entail weighing up life and death.

The EQ-5D-Y is a concise, generic measure of child HRQoL [7]. It was adapted from the EQ-5D, a measure of adult HRQoL recommended in 85% of HTA methods guides [8]. The EQ-5D-Y is validated for use in 8–15 year olds [9] via self-report or proxy completion [10]. The EQ-5D-Y is intended to be accompanied by value sets to facilitate its use in HTA in the same manner as its adult counterpart. Following the launch of EQ-5D-Y in 2010, nearly a decade passed until a protocol was made available for the development of value sets for it. During that time, the EuroQol Group undertook a programme of methodological research to inform methods choices. That work culminated in publication of an international protocol to guide valuation studies [11] enabling a first wave of studies to proceed. While some methods issues remained unresolved, it was considered there was sufficient evidence to support the methods chosen; notably, the protocol was not prescriptive about all aspects of methods (as explained in the next section). It was anticipated that the first valuation studies would provide further insight into how the protocol performed, to identify any adaptions required to improve it.

Use of EQ-5D-Y had been gradually increasing, generating demand for value sets to use in analysing those data. Strong interest in producing EQ-5D-Y value sets, combined with the availability of a protocol and support from the EuroQol Group, led to the rapid development of value sets. To date, four value set studies have been published: Slovenia [12] Japan [13] Spain [14] and Germany [15]. Seven other studies are underway and close to completion (Netherlands, Belgium, Australia, Hungary, mainland China, Hong Kong, and Indonesia) while six other studies have recently commenced or are preparing to commence soon (including the US, Brazil, Malaysia, Singapore, Taiwan and Vietnam). By the end of 2022—just two years after publication of the protocol—between 11 and 17 EQ-5D-Y value sets are likely to be available, more than any other child HRQoL measure [4].

The rapid uptake of the protocol means that it is timely to review the experience of those using it; to ensure that the research community benefits from any learnings from the work to date; and to identify remaining issues where more research is needed.

In June 2021 the EuroQol Group organised a three-day workshop, bringing together all those involved in country-specific EQ-5D-Y value set studies and related methodological research concerning EQ-5D-Y and valuation. The aims of the workshops were to (a) evaluate initial experience using the EQ-5D-Y valuation protocol, (b) identify what further methods research or other efforts (e.g. stakeholder engagement) are required to strengthen EQ-5D-Y valuation and to support users of EQ-5D-Y values e.g., in estimating QALYs.

The aim of this paper is to briefly describe the state of play with EQ-5D-Y valuation and to provide an overview of the workshop deliberations. We highlight areas of consensus and ongoing debate about the methods and procedures for valuing EQ-5D-Y; and the conclusions which have been drawn from the workshop, including implications for future research, and issues for end users to consider.

We begin by providing a brief summary of the current EQ-5D-Y valuation protocol and studies underway using it. We then describe the workshop and the means by which we recorded and summarised the discussions that took place. A narrative summary and synthesis of those discussions is provided, together with conclusions which have been drawn from them.

## EQ-5D-Y valuation protocol and use to date

Table 1 summarises the principal elements of the EQ-5D-Y valuation protocol [11]. It includes both discrete choice experiments (DCE) and time trade off (TTO), specifically the composite TTO (cTTO), as is also used for valuing EQ-5D-5L [16].

**Table 1:** International valuation protocol for EQ-5D-Y: key elements of the methods

Protocol feature	DCE	cTTO
Whose values are relevant?	Adult general population	Adult general population
What perspective?	Hypothetical child	Hypothetical child
Age of child?	10 years of age	10 years of age
Duration of states?	Not specified	10 years
Design?	10 blocks; 15 pairs each respondent	1 block of 10 states
Specific format of task?	Pairwise choice; no indifference option	Composite TTO i.e. conventional TTO for values > 0, lead time TTO for values < 0
Sample size?	1000	200
Mode of administration?	Online self-completion	Computer-assisted personal interviews (CAPI), conducted either in person or online
Number of tasks per respondent?	15 pairwise choice tasks	10 cTTO tasks

However, there are notable differences between the valuation protocols for EQ-5D-5L and EQ-5D-Y:

- The role of DCE and cTTO is different. When valuing EQ-5D-5L health states, DCE and cTTO are used as complementary methods and both are administered to all respondents via computer assisted personal interviews (CAPI). When valuing EQ-5D-Y health states, DCE is the principal method used to determine the relative importance of dimensions and severity levels. The DCE tasks are relatively simple and involve asking participants to indicate which of two health states they prefer. However, DCEs yield results on a latent scale i.e., the resulting preferences are measured in terms of unanchored utility with no units. Thus, some means of anchoring results to dead = 0 and full health = 1 is required if the value sets are to be used to construct QALYs and that is the role of cTTO in the EQ-5D-Y valuation protocol [17, 18]. The DCE and cTTO data can be used in different ways to model EQ-5D-Y value sets, and the protocol is not prescriptive in this respect. For example, the cTTO data can be used to anchor the DCE data by (i) using just the mean cTTO value of the worst state to ‘rescale’ the DCE values, (ii) using ‘mapping' modelling approaches, based on the relationship between observed *mean c*TTO values and DCE values, and (iii) using ‘hybrid’ modelling approaches that take account of all *individual*-level DCE and TTO observations [19].
- While both protocols involve having adults value the health states defined by the EQ-5D-5L and EQ-5D-Y, there is a difference in the perspective adopted. In the case of EQ-5D-Y, adults are asked to value health states which may be experienced by someone else (specifically, they are asked to consider their views about a 10 year old child), while in the case of EQ-5D-5L (or EQ-5D-3L) health states, they are asked to imagine experiencing those states in themselves.
- In the EQ-5D-5L protocol all respondents complete both cTTO and DCE tasks in CAPI interviews. In contrast, the EQ-5D-Y protocol suggests using two separate samples: a larger sample self-completes the DCE tasks online, capitalising on the advantage of this being a quick and less resource-intensive means of data collection, while a smaller sample completes interviewer-administered cTTO tasks either in person or online.^1^ The cTTO tasks entail an iterative questioning process that is not well suited to self-completion.

The value set studies which are completed, underway or planned are shown in Table 2. There are differences between these studies’ use of cTTO data to create a final value set; some used the mean observed or modelled cTTO value for the worst health state defined by the EQ-5D-Y (33333), while others used a mapping function to link DCE and cTTO data.

**Table 2:** Overview of EQ-5D-Y valuation studies which are published, data collection completed, underway

Country/Region	Status of EQ-5D-Y value set	Departures from the protocol?	Additional Methodological research	How are values anchored?
Japan	Published—see Shiroiwa et al. [13]	cTTO designs with five blocks each with six health states; Same sample completed both DCE and cTTO tasks in face-to-face interviews		Mapping DCE data onto the cTTO data
Slovenia	Published—see Prevolnik-Rupel et al. [12]	Standard	DCE included an adolescent sample aged between 11 and 17 years [23]	Rescaling based on the single state “33333”
Spain	Published—see Ramos-Goni et al. [14]	cTTO tasks switched from face-to-face to online settings part-way through	DCE included an adolescent sample aged between 11 and 17 years [23]	Rescaling based on the single state “33333”
Germany	Published—see Kreimeier et al. [15]	cTTO tasks switched from face-to-face to online settings part-way through	DCE also included in cTTO interviews to compare interview-based and online DCE results; DCE included an adolescent sample aged between 11 and 17 years [23]	Mapping DCE data onto the cTTO data*
Belgium	Data collection completed	cTTO tasks switched from face-to-face to online settings part-way through	Additional DCE respondents were included, whom completed tasks from the perspective of a child (8–12 years old) and adolescents (13–18 years old)	Rescaling based on the single state “33333”*
China (Mainland)	Date collection completed	Expanded cTTO design to allow possibility of modelling cTTO-only value set (a small orthogonal design with 18 states + 10 from protocol)		Methods to be determined
Hong Kong	Date collection completed	both DCE and cTTO tasks administered in face-to-face CAPI	DCE and cTTO tasks using two perspectives: a child (10 year old) and adult’s own perspective	Methods to be determined
Hungary	Data collection completed	Standard	Additional four TTO tasks using adult’s own perspective	Rescaling based on the single state “33333”*
Indonesia	Data collection completed	Expanded TTO design (18 health states arranged orthogonal design + 5 suggested states + 2 states with higher level sum score)		Mapping DCE data onto the cTTO data*
		Paper-and-pencil survey for DCE		
Netherlands	Data collection completed	Expanded TTO design to allow possibility of modelling TTO-only value set (28 states divided into 3 blocks of 10 stats each; 33333 and at least one mild state in each block); cTTO tasks completed in online settings	An additional cTTO sample (n = 203) was collected, in which the cTTO task was framed asking respondents to indicate what they think a 10 year-old child would prefer	Different approaches used, including (1) Rescaling based on the single state “33333” and (2) mapping DCE data onto cTTO data
Australia	Underway	Expanded cTTO design to allow possibility of modelling cTTO-only value set; cTTO tasks planned in online settings	Additional DCE with duration and DCE with dead tasks	Different approaches planned:
				(1) Rescaling based on the single state “33333”; and using other health states in cTTO as the sole anchor
				(2) Mapping DCE data onto cTTO data
				(3) using DCE with duration; (4) using DCE with dead questions
US	Underway	Preparatory work underway		
Brazil	Underway	Preparatory work underway		
Malaysia^+^	Underway	In all four cases, an expanded cTTO design to allow possibility of modelling cTTO-only value set (a small orthogonal design with 18 states + 10 from protocol)	A pilot DCE study of 400 individuals in each country/region, who will be randomised to the perspective of a hypothetical child from the following age groups: “5–7 years old”, “8–10 years old”, “11–13 years old” and “14–15 years old”	Different approaches planned:
Singapore^+^	Underway			
Taiwan^+^	Underway			(1) Rescaling based on the single state “33333”
Vietnam^+^	Underway			(2) Mapping DCE data onto cTTO data
				(3) a hybrid model

^*^For unpublished studies, we list here the anchoring method reported at the workshop, based on preliminary analysis

^+^Four different value sets for Malaysia, Singapore, Taiwan and Vietnam will be generated separately, but they follow the same study design and methodological add-ons

Some of the studies departed from the published protocol, primarily in two ways. First, by extending the number of states included in the cTTO tasks (e.g. Australia, China, Indonesia, Netherlands, Malaysia, Singapore, Taiwan, Vietnam). While the number of states suggested for cTTO valuation in the original published protocol was small, that was the minimum recommended number and was intended to allow estimation of a value for ‘dead’ and to make initial studies as feasible as possible. Experimentation with larger sets of states within the cTTO protocol was encouraged, however, where considered feasible by local teams. Second, while the protocol anticipated that CAPI interviews would be conducted face-to-face, the COVID-19 pandemic led to an increased use of online interviews.

Some of the studies added methodological components in their value set studies to explore alternative valuation methods (e.g. DCE with duration and DCE with dead choice tasks) [20–22]; the possibility of eliciting preferences from adolescent samples [23]; and different perspectives (e.g. adult respondents asked to imagine experiencing the EQ-5D-Y health states in themselves, rather than for a 10-year old child; using the perspective of a hypothetical child in different age groups) [24, 25]. These methodological add-ons (listed in Table 2) are encouraged by the EuroQol Group, alongside the standard protocol, to maximise learning opportunities.

In addition to the studies completed or underway, as described in Table 2, further value set studies have been proposed in key countries with well-established HTA systems.

## Methods: workshop organisation and methods used to record discussion

The workshop was held online over three consecutive days. Day 1 focussed on reporting the value set studies which were either completed or underway. Each principal investigator (PI) was invited to submit an i-poster summarising their experience of implementing the protocol, addressing: (a) engagement (if any) with local HTA bodies; (b) methods/design (if ‘non-standard’); (c) sample and data quality; (d) value set modelling choices; (e) key findings and; (f) issues arising. Posters were circulated to participants in advance, and two discussants reviewed them and presented a synthesis of the results to the workshop. Comparative analysis of emerging (as yet unpublished) values across countries was undertaken and reported, with an emphasis on identifying similarities or differences.

Day 2 focused on updates from methodological work on EQ-5D-Y valuation, including both experimental research to test alternative methods, and theoretical and normative considerations regarding eliciting and interpreting values for child HRQoL.

Day 3 provided a summary of the previous days’ discussions, before breaking into small focus groups facilitated by the authors. Focus groups were asked to consider four questions, developed by the authors in the light of the previous days’ discussions: (a) Is the current EQ-5D-Y valuation protocol fit for purpose? What is missing; what needs changing? (b) Before commencing EQ-5D-Y value set studies, should research teams engage with HTA and other stakeholders? (c) What are the top priorities for methodological research in the field of EQ-5D-Y valuation? (d) Is a one-size-fits all protocol required, or can methods vary between countries?

Invitees (listed in full in the acknowledgements) were 37 individuals (including the authors) identified as having an active research interest in valuation of EQ-5D-Y and/or having other relevant expertise in HRQoL valuation and were primarily but not exclusively members of the EuroQol Group. All those who were invited attended, apart from one person where the time difference made this difficult. Attendance was high, with nearly all invitees attending throughout the three days, and very active participation in the sessions.

The workshop was captured by recording all sessions, saving chat box entries and note taking (TP). Consent to record was obtained from workshop participants in advance. A narrative overview of each day’s discussions, based on all three sources, was produced by TP and ND. Identification of key points of consensus and debate were produced by ND using the narrative overview and consulting online chat records. The overview and key points were reviewed by all authors. Where interpretation of any participants’ comments was unclear, we checked the recording and followed up with individual participants to clarify. A report containing the summary and conclusions was circulated to all participants.

## Results from workshop discussions

In this section we provide a high-level summary of discussions that took place over the three days, organising these around the questions posed to focus group participants on Day 3.

### Is the current EQ-5D-Y valuation protocol fit for purpose? What is missing; what needs changing?

There was consensus that the DCE component of the protocol is ‘working well’. Opinions were divided about the best way to anchor DCE values to a 0 = dead scale. However, there was consensus that it is preferable to avoid using the cTTO solely to estimate a value for the worst health state defined by EQ-5D-Y (state 33333), because using all available cTTO data allows for more precise estimates of values for states 33333 and adjacent states.

There was agreement that, given learning from the initial studies, it is preferable to err towards the use of somewhat larger sub-sets of EQ-5D-Y health states in the cTTO exercise to better support hybrid modelling, especially if other modelling approaches (e.g. producing cTTO-only value sets^2^) are preferred.

There was a broad consensus that, although the preferences of the general public are generally considered most relevant for HTA, evidence on the preferences of adolescents for the EQ-5D-Y may be relevant to some users of value sets and that this could form an active line of research. There was debate about whether *separate* value sets for adolescents and adult preferences are required for some purposes; or whether adolescents’ views should be combined with adult preferences for establishing a final value set. Related discussion included whether, for ethical reasons, adolescents should only be asked to participate in the DCE tasks or whether it is also reasonable to ask them to complete cTTO tasks involving life/death trade-offs.

There are a wide range of views about the best methods to use to elicit stated preferences for child HRQoL. Even among those who advocate use of TTO, there are differing views around which specific type of TTO is most appropriate for valuing EQ-5D-Y health states.^3^ There have been concerns that cTTO methods could produce higher values for the EQ-5D-Y than for comparable adult EQ-5D states. This finding, from a multi-country pilot of EQ-5D-Y valuation that informed the protocol, and supported by more recent research [28], has been linked to adults’ unwillingness to trade off life years for a child [17]. This issue is not specific to cTTO but could potentially affect any method that involves trading off survival and quality of life in children. The resulting differences in value scale length could affect the comparability of child and adult values used in estimating QALYs [13, 29]. The emerging evidence on this from studies using the protocol are somewhat mixed—there is some evidence pointing to higher values (e.g. the Japanese value set for the Y-3L contains *no* negative values [13]) and to adults’ unwillingness to trade for children [30]. However, there is also evidence suggesting unwillingness to trade-off survival for children is not absolute, but depends on the dimensions and severity of states [31]. Further research is needed to understand how value sets for child and adult instruments compare.

A key remaining issue is how best to anchor latent scale DCE data. In particular, whether to continue with combining DCE and cTTO data (in keeping with the current protocol, but with an amended TTO design) or to introduce new methods—for example, variants of DCE which include comparisons of states with dead, or that include duration as one of the attributes [32] or other new methods being developed (for example, personal utility functions) [33, 34]. Until innovative methods are demonstrated to have superior properties, however, the protocol for valuing EQ-5D-Y is expected to continue to be based on the combination of DCE and cTTO.

Participants noted concerns about the potential lack of direct comparability of EQ-5D-Y value sets and value sets for adult versions of the instrument. As noted above, values for health problems in children tend to exceed the values for corresponding adult HRQoL states, creating discontinuity in values (e.g., when applied in economic evaluation models that involve transitions from childhood to adulthood). This also has implications for the use of EQ-5D-Y values in HTA, where estimates of child QALYs and adult QALYs are assumed to be comparable. The issue arises from adults valuing child states, and prioritising child survival over child quality of life. It is important to note that this problem may not just apply to TTO: *any* method anchoring at dead = 0 (for example, standard gamble, or DCE with duration) might encounter the same issues with respect to adults’ unwillingness to trade child life leading to scale compression.

### Before commencing EQ-5D-Y value set studies, should research teams engage with HTA and other stakeholders?

The workshop revealed there had been little active engagement with HTA bodies so far on EQ-5D-Y valuation methods, despite the implications of methods choices for results. Notable exceptions include Belgium where considerable efforts are being made by PIs to consult with local decision makers. Detailed stakeholder engagement is currently underway in the US and Canada, in preparation for future value set studies.

There was agreement that it is desirable to more actively engage with HTA bodies and other decision makers to a) ensure that they are aware of the methods used to value EQ-5D-Y health states, and b) ensure those choices align with local user requirements. As noted above, the development of EQ-5D-Y value sets involves a divergence from the methods commonly used to produce value sets for adult EQ-5D instruments. It is important that decision-makers are aware of those differences and the implications for interpreting and using values.

There was some uncertainty about how to proceed if HTA bodies lack awareness of the issues around valuing child HRQoL; or are aware of the issues but lack any clear views on preferred methods or on the normative principles underpinning methods choices. Stakeholder engagement may therefore need to include raising awareness of the relevant issues. While some considered that consultation and engagement should be a required phase in *all* EQ-5D-Y value set studies, an alternative view is that efforts should focus on HTA bodies who are likely to be the best informed and have internal processes for dealing with methods issues e.g., NICE (UK).

### What are the top priorities for methodological research in the field of EQ-5D-Y valuation?

There was a general consensus that addressing methods for anchoring DCE data on a QALY scale with dead at 0 and full health at 1 is a priority. A first step is to strengthen the design of the states included in the cTTO tasks in the protocol. The fact that some of the studies have chosen to depart from the protocol by increasing the number of health states in cTTO tasks indicates the clear need to improve this component. One consideration when exploring methods regarding anchoring is whether and how these preferences should be elicited from adolescents.

### Is a one-size-fits-all protocol required, or can methods vary between countries?

There was a general consensus that there is no single, ‘correct’ normative basis for valuing EQ-5D-Y, and that there never will be a set of methods that are appropriate for all users and potential applications of these values. This issue is closely linked with the need for engagement with HTA bodies and other end users, noted earlier, to ensure values are ‘fit for purpose’.

There were mixed views about how to balance flexibility (e.g. to meet local user requirements) with comparability between value sets (a key advantage of standardised EuroQol valuation protocols). While there is room for experimentation alongside the core protocol, and modifications are possible if needed to align with local stakeholder requirements, it is desirable to stay as close to the core protocol as possible to allow for comparability across value sets.

## Discussion

Most of the EQ-5D-Y value set studies considered in the workshop are yet to be published, so a limitation of this summary of the workshop is that we are unable to report quantitative results from them. However, the discussions reported here capture all important aspects of both the emerging values and the experiences of the PIs in using the protocol, providing a basis for early evaluation of its performance. The workshop was able to identify aspects of valuation methods where there is consensus—and the remaining areas of disagreement and actions needed to address that.

The workshop has helped to identify clear priorities for further methods research. It is important that both study teams and users of value sets be aware that the current valuation protocol, rather than being definitive, is anticipated to evolve as we benefit from the experiences and data generated from both the first wave of studies, from ongoing methods work (e.g., on anchoring) and from work to test the psychometric properties of EQ-5D-Y data summarised by the emerging value sets.

Given remaining questions about methods, and the role of value judgements in methods choices, it was concluded that stakeholder engagement should be regarded as a key part of future value set studies. HTA bodies may lack clear views on these issues [3], so consultation may require efforts to raise awareness and understanding of the relevant issues. Reflecting local stakeholder principles and views into research designs needs to be carefully balanced against potential loss of comparability between countries. However, while the EuroQol Group has a specific methodology it recommends for valuing EQ-5D-Y health states, it does not impose restrictions on the methods to be used in valuing its instruments, and welcomes innovation. Other than providing access to its protocols and expertise, and ensuring the implementation of its quality control process, the EuroQol Group does not impose any process for approving (or not) value sets [32].

### Implications for users

The value sets being produced for EQ-5D-Y meet a need to summarise EQ-5D-Y data, e.g., for statistical purposes**.** EQ-5D-Y values are also appropriate to use in producing estimates of QALYs and QALY gains in children, to compare HRQoL and QALYs between different groups of children and to assess the relative effectiveness and cost effectiveness of interventions for children.

However, it is important to note that values for the EQ-5D-Y and values for adult EQ-5D instruments may not be directly comparable. Concerns about comparability arise both because of the difference between the task of valuing own health as opposed to health in someone else, and because of potential unwillingness of adult respondents to sacrifice life years in a child affecting their TTO responses [24, 29]. This makes it potentially inappropriate to assume that a QALY gained or lost for an adult is the same as a QALY gained or lost for a child. This is particularly important where such evidence is used in HTA, where comparability of QALY estimates is assumed. As noted in Shiroiwa et al. [13] **“**Because the QALY weights for children are higher than the QALY weights for adults, a similar degree of improvement in an adult’s health state will generate fewer QALYs for a child. Thus, simple comparisons of cost-effectiveness ratios for adults and children should be avoided”.

A priority for further research is how to address any discontinuities in the value scale that arise e.g. in applications that involve a transition from utilities for EQ-5D-Y to adult EQ-5D instruments. It is important to note that these issues with scale length and non-comparability are potentially an issue with values for *all* paediatric HRQoL instruments—but the EuroQol Group is in a unique position to address it, because the EQ-5D-Y has a conceptual relationship with the (adult) EQ-5D—something which other paediatric HRQoL instruments lack.

There remain a number of assumptions underpinning the valuation of EQ-5D-Y. For example, similar to values for the EQ-5D adult instruments, the *duration* of states to be valued using cTTO is fixed at 10 years. The use of these values to quality-weight states with much shorter durations relies on the assumption of constant proportionality, which may not hold [35, 36]. This issue is not specific to valuation of child HRQoL. However, it is further complicated in the case of valuing EQ-5D-Y states, since the 10 year duration combined with the age of the hypothetical child means that the 'lives’ being assessed in the cTTO tasks extend into adulthood (20 years of age where states are better than dead; 30 years of age where states are worse than dead). Further, the use of these values assumes that the values can be applied to EQ-5D-Y data obtained from children both much older and much younger than 10 years of age. A number of valuation studies underway or planning (e.g. US, Malaysia, Singapore, Taiwan, Vietnam), plan to examine the impact of changing the age of the hypothetical child as part of their studies. Further research to explore the effect of age and duration on values is needed to understand the importance of these assumptions for users.

## Conclusions

The availability of a protocol for valuing EQ-5D-Y has facilitated rapid production of value sets. The availability of these is key to increased use of EQ-5D-Y to help address substantial evidence gaps in evidence on child HRQoL.

Methodological questions inevitably remain and the workshop has identified research priorities. The acceptability of methods and resulting values to HTA bodies and other stakeholders is important, and future studies will incorporate efforts to engage with end users.

The protocol, and the studies currently underway using it, focus on the three level version of the EQ-5D-Y (i.e. EQ-5D-Y-3L). A five-level version of the instrument is available (EQ-5D-Y-5L) [37], but is currently considered an experimental version. Nevertheless, a growing body of psychometric evidence exists to support the EQ-5D-Y-5L moving beyond experimental status [38–40], and value sets to accompany it will be required. The issues discussed in this paper are equally relevant to the EQ-5D-Y-5L.

Finally, it is important to note that many of the issues surrounding valuation of EQ-5D-Y instruments are not unique to it: the same challenges are encountered in valuing other measures of child HRQoL. All paediatric value sets are based on judgements about whose preferences are relevant and what methods are best to elicit them. There is no standard approach to valuation for any of the child HRQoL measures [6]. The EuroQol Group is committed to transparency about empirical and normative issues relating to valuation, and to the use of emerging evidence from methodological studies to strengthen its valuation protocol.

## Data Availability

Not applicable.

## Abbreviations

HRQoL: Health-related quality of life
HTA: Health Technology Assessment
QALYs: Quality adjusted life years
TTO: Time trade off
DCE: Discrete choice experiments
CAPI: Computer assisted personal interviews

## Footnotes

1 In some EQ-5D-Y studies DCE and TTO data are collected together – e.g. Japan [13].

2 Seven studies indicated they had expanded or planned to expand the number of states included in cTTO tasks in order to allow the possibility of modelling a cTTO-only value set.

3 For example, using cTTO to value EQ-5D-Y health states considered worse than dead can lead to a paradoxical scenario, whereby the ‘valuer’ is asked to value a state for a 10-year-old child by imagining the child spends 10 years in full health, followed by 10 years in the state being valued. In that scenario, the state being valued is therefore actually being experienced by a 20 – 30-year-old, rather than by a child. Alternative TTO approaches, such as lag time TTO – where the additional trading time in full health follows the poor health state being valued, rather than preceding it as in lead time TTO which is used to value states worse than dead in the cTTO, may have some advantages in this regard but also have other limitations [26, 27].
